# Control of Invasion by Epithelial-to-Mesenchymal Transition Programs during Metastasis

**DOI:** 10.3390/jcm8050646

**Published:** 2019-05-10

**Authors:** Gray W. Pearson

**Affiliations:** Lombardi Comprehensive Cancer Center and Department of Oncology, Georgetown University, Washington, DC 20057, USA; gp507@georgetown.edu; Tel.: +1-202-687-0807

**Keywords:** metastasis, epithelial-to-mesenchymal transition, collective invasion, heterogeneity, hybrid

## Abstract

Epithelial-to-mesenchymal transition (EMT) programs contribute to the acquisition of invasive properties that are essential for metastasis. It is well established that EMT programs alter cell state and promote invasive behavior. This review discusses how rather than following one specific program, EMT states are diverse in their regulation and invasive properties. Analysis across a spectrum of models using a combination of approaches has revealed how unique features of distinct EMT programs dictate whether tumor cells invade as single cells or collectively as cohesive groups of cells. It has also been shown that the mode of collective invasion is determined by the nature of the EMT, with cells in a trailblazer-type EMT state being capable of initiating collective invasion, whereas cells that have undergone an opportunist-type EMT are dependent on extrinsic factors to invade. In addition to altering cell intrinsic properties, EMT programs can influence invasion through non-cell autonomous mechanisms. Analysis of tumor subpopulations has demonstrated how EMT-induced cells can drive the invasion of sibling epithelial populations through paracrine signaling and remodeling of the microenvironment. Importantly, the variation in invasive properties controlled by EMT programs influences the kinetics and location of metastasis.

## 1. Introduction

**Figure 1:**
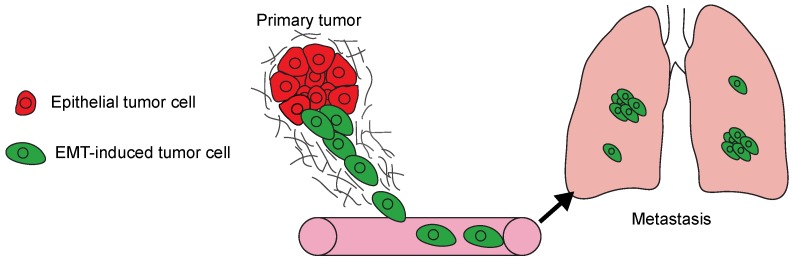
The model summarizes the steps involved in the development of metastasis. Epithelial-to-mesenchymal transition (EMT) program activation in tumor cells (green) promotes local invasion. The invasive cells intravasate into blood vessels and disseminate to new tissues, in this case the lungs. Disseminated tumor cells then initiate colonizing metastatic growth in the new organ.

The acquisition of invasive ability (Figure 1) is an essential first step towards the development of metastatic cancer [1]. After invading into the connective tissue, tumor cells can intravasate into blood vessels and disseminate to new tissues [2]. Early attempts to define the properties of invasive tumor cells revealed that tumor cell cohesion is reduced relative to the tissue of origin [3], and that tumor cells could migrate as solitary cells or as multicellular groups in culture [4]. Notably, it was recognized that the duration of the tumor growth and the number of tumor cells entering the blood stream correlated with the extent of metastasis [5]. These collective findings have suggested that alterations to cell features that promote dissemination contribute to metastasis. 

The acquisition of invasive traits by tumor cells mirrors the phenotypic changes of epithelial-to-mesenchymal transitions (EMTs) that take place during embryogenesis and wound healing [6]. The EMT process involves a loss of polarity, a disruption of cell–cell adhesion, and the acquisition of migratory ability [7]. These changes in cell state are coordinated by a combination of secondary modifications to existing proteins and alterations to cell signaling pathways through transcriptional and post-transcriptional changes that alter the pattern of gene expression [8]. Given that the properties of developmental EMT programs mirror essential features of invasive tumor cells, processes that regulate EMTs have been investigated in the context of neoplastic cell behavior [9]. Importantly, advances made in unravelling the regulation of EMTs that contribute to tissue development and inflammatory responses have established a signaling framework that has been used to reveal that EMTs contribute to tumor invasion and metastasis [10].

## 2. EMT Program Regulation and Function

Epithelial tissue is comprised of adherent polarized sheets of cells that, depending on tissue type, are sculpted into ducts and lobules [11]. Tumors initially proliferate within luminal spaces and are separated from the stromal compartment containing conduits of metastasis [12]. The durable cell–cell attachments formed by normal and tumor cells within these lesions prevent spontaneous movement and invasion [13]. As is observed during embryogenesis and tissue morphogenesis, EMTs in tumor populations promote invasion by triggering a loss of polarity and cellular cohesion, while also conferring migratory properties and the ability to reorganize the extracellular matrix (ECM) [14].

### 2.1. Mechanism EMT Program Activation

EMT programs are normally initiated by ligands that bind to transmembrane receptors capable of activating intracellular signaling pathways. Examples include members of the TGFβ family, growth factors that bind to receptor tyrosine kinases (RTKs), and WNT ligands [15]. These signaling cues are expressed in the tumor microenvironment by recruited fibroblasts and leukocytes, which create niches where tumor cells undergo EMTs [16]. Genetic abnormalities also contribute to tumor cell EMTs, as evidenced by the ability of tumor cells to sustain mesenchymal features in the absence of extrinsic signaling cues from non-tumor populations [17]. The signaling pathways coordinated by these various receptors share the general feature of activating transcription factors that induce the expression of the core EMT transcription factors (EMT-TFs), Snail, Slug, Twist, Zeb1, and Zeb2. These EMT-TFs then directly repress epithelial cell–cell adhesion and polarity genes, while also inducing mesenchymal factors that alter the organization of the cytoskeleton, contribute to protrusion formation, and modulate the rate of cell migration. The induction of EMT programs is influenced by cell lineage-associated microRNAs (miRNAs), including miR200a, miR203, and miR205, which directly target EMT-TFs to restrict expression [18]. Additional layers of regulation include differential splicing and post-translational modifications that enhance EMT-TF stability, and epigenetic modifications that control chromatin accessibility [19]. Biomechanical feedback also influences EMT program transcription through control of EMT-TF subcellular localization [20].

### 2.2. Suppression of Epithelial Traits

The most established mechanism by which EMT programs promote cell migration is the suppression of the cell–cell adhesion protein E-cadherin. Reduced E-cadherin expression correlates with poor patient outcome [21,22,23,24] and is associated with enhanced invasive traits and metastatic capability [25]. Snail, Slug, Zeb1, and Zeb2 directly bind E-Box recognition sites in the E-cadherin promoter [26] and recruit histone methyltransferases, demethylases, and deacetylases to create a repressed chromatin architecture that drastically reduces or eliminates E-cadherin expression [27]. The loss of E-cadherin in transformed mammary epithelial cells is sufficient to promote the further induction of an EMT, invasion, and metastasis [28], highlighting the critical function of E-cadherin in sustaining epithelial fidelity. However, the loss of E-cadherin alone is not able to induce an EMT in all contexts [29], as it is typically a downstream signaling event coordinated by a more elaborate EMT program. As an alternative to transcriptional silencing, E-cadherin can be subjected to an increased rate of recycling by endocytosis [30,31], with the net effect of allowing transient adhesion formation that permits migration while retaining cell–cell cohesion [32]. In addition, cell cohesion is reduced by EMT-TF mediated suppression of proteins that contribute to tight junction, gap junction and desmosome formation [33]. The destabilization of cell–cell junctional integrity induced by EMTs also causes a disruption in the establishment of adhesion-associated PAR and the Crumbs polarity complexes [34]. Loss of apical-basal polarity is further reinforced by the suppression of polarity protein expression [35]. 

### 2.3. Induction of Mesenchymal Features

The loss of epithelial features alone is not sufficient to promote migration and invasion. EMT-TFs also induce mesenchymal genes that promote alterations in cell morphology, enhance migratory properties, and influence the ability to remodel the ECM [7]. EMTs also confer cells with the capacity to form protrusive structures and acquire a bipolar morphology [36] through the induction and alternative splicing of genes that regulate localization and duration of actin polymerization [37,38]. The induction of the intermediate filament protein vimentin is a canonical feature of EMT programs that is frequently used as a marker of cells that have undergone an EMT [39]. Tissue-specific keratin expression is also suppressed as cells progress to a more fully mesenchymal state [39]. One of the consequences of this change in intermediate filament composition is a perturbation in protein trafficking and interactions with motor proteins [27]. Cell substrate adhesion proteins and receptor composition are also altered to change the stability and duration of adhesive structures and how cells respond to new ECM niches [40]. These changes in cell morphology allow EMT-induced cells to respond to chemotactic signals and migrate through existing tracks in the ECM created by non-tumor populations in the microenvironment [41]. EMT programs also endow tumor cells with the ability to remodel the ECM themselves. The induction of matrix metalloproteinases, which cleave basement membrane proteins and collagens, facilitates the initial invasion from ductal structures, migration through stromal tissue, and intravasation into blood vessels [42]. The composition and adhesive properties of the ECM can be further altered through secretion of proteins such as fibronectin and Tenascin-C [43,44].

## 3. EMT Program Heterogeneity Confers Distinct Invasive Phenotypes

**Figure 2:**
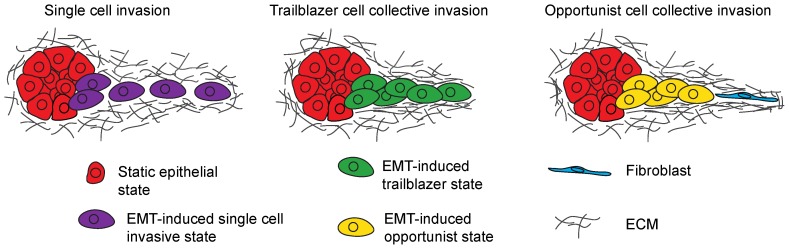
Model shows the different modes of invasion induced by EMT programs. Tumor cells can engage in single cell invasion (purple), trailblazer type collective invasion (green) or opportunistic collective invasion (yellow) depending on the nature of the EMT program that is activated.

There is heterogeneity in the composition and functions of EMT programs. The elements of epithelial suppression and mesenchymal induction just described are not a part of a single EMT program through which cells progress over time. Thus, it should not be assumed that a feature of one EMT program is a trait of all EMT programs. There are a range of unique EMT states, reflecting distinct activating signals and intrinsic cell-lineage features, that determine the extent of epithelial gene suppression and mesenchymal gene induction that occurs as part of an EMT program. This heterogeneity in EMT programs contributes to the significant phenotypic variability observed in the modes of tumor cells invasion [45] (Figure 2). 

### 3.1. Regulation of Single-Cell Invasion

Tumor cell invasion is frequently conceptualized as a process undertaken by solitary cells that detach from a multicellular tumor mass and migrate into the ECM. There are distinct modes of single-cell invasion [46]. Cells can engage in a mesenchymal mode that is dependent on the activity of proteases, such as matrix metalloproteinases, that degrade ECM proteins [47]. Tumor cells can also migrate using force-dependent cytoplasmic blebbing to push through gaps in the ECM, independent of protease activity [48,49]. A more fully mesenchymal state characterized by E-cadherin suppression and vimentin induction is associated with the ability of tumor cells to dissociate and invade as individual cells [17,50,51]. However, it should be noted that there is evidence suggesting that the retention of epithelial traits, such as E-cadherin expression, does not preclude the induction of single-cell invasion and may be promoted by a hybrid EMT state [52,53,54]. Single-cell invasion can be induced by a range of signals, including TGFβ [55], CXCL family chemokines, RTK ligands, and hypoxia [56]. Single-cell invasion is a relatively rare event in primary tumors and is most frequently detected proximal to blood vessels [51]. Intravital imaging has revealed that single cells can move rapidly along pre-existing aligned ECM fibers that act as paths towards blood vessels [57]. Evidence of both mesenchymal and rounded or ameboid modes of single-cell invasion is detected in EMT-induced cells [51]. In addition, EMT-induced tumor cells can convert between mesenchymal and ameboid modes of migration spontaneously, or in response to changes in ECM composition or experimental intervention [45]. The extent to which EMT programs directly control a switch between modes of single-cell invasion is not known.

### 3.2. Collective Invasion Is the Predominant Mode of Tumor Cell Invasion

Tumor cells frequently engage in a process called collective invasion, in which cells migrate through the ECM in groups of cells that retain cellular cohesion [58]. During collective invasion a leading tumor cell extends protrusions that establish traction and exert force on the ECM [59]. These protrusions also secrete proteases to further promote ECM degradation [60]. Additional cells track along the paths created by the first leading cell [61], widening the path in the ECM, and allowing the parallel invasion of cells [62]. Importantly, collective invasion is the principal mode of tumor invasion, as determined by the reconstruction of the primary tumor organization [63], evaluation of tumor explants [64,65], and intravital imaging [54,66]. There is variability in the mode of collective invasion induced by EMT programs. One class of EMT programs confers a trailblazer phenotype that is characterized by an enhanced ability to initiate collective invasion [67,68,69]. A second class of EMT programs induces an opportunistic state in which cells are motile, but are dependent on extrinsic factors, such as the recruitment of fibroblasts, to collectively invade [70,71].

### 3.3. Trailblazer-Type Collective Invasion

The trailblazer EMT program is distinguished by the induction of genes that are specifically required to form cellular protrusions that provide traction and reorganize collagen into parallel fibrils [68,72]. These trailblazer-specific proteins include DOCK10, a guanine nucleotide exchange factor that activates Cdc42 [73], integrin α11, a collagen 1 specific integrin [74], DAB2, which contributes to integrin endocytosis [75], and PDFGRA, which activates signaling pathways necessary for ECM degradation [76]. These proteins coordinate distinct pathways that are integrated together to promote this highly invasive phenotype [68]. Cells with trailblazer features also secrete fibronectin and express higher levels of vimentin [69]. Trailblazer cells investigated to date lack E-cadherin expression, yet retain cellular cohesion [68,69]. A switch from E-cadherin to N-cadherin expression is a feature of some EMT programs [77], and thus potentially provide a mechanism for trailblazer cells to retain cell–cell attachments. In squamous carcinoma models, the cohesion of trailblazer-type cells is sustained by Snail-dependent expression of the tight junction protein Claudin 11 [78]. Genes required for trailblazer cell collective invasion are also necessary for metastasis [68], suggesting that the intrinsic ability of cells to initiate collective invasion influences dissemination.

### 3.4. Opportunistic-Type Collective Invasion

Opportunistic EMT states can be induced by hybrid programs that confer mesenchymal features while allowing cells to retain epithelial character. Hybrid states are a general property of carcinomas [79] and collectively invading hybrid tumor cells are detected in breast, lung, and pancreatic patient tumors [63]. Live-imaging of 3-dimensional culture systems has revealed that cells in a hybrid state are motile within spheroids, yet are unable to initiate invasion into the ECM [70,80,81]. The opportunistic nature of hybrid EMT migration can also be inferred by contrasting the ability of these cells to collectively migrate in wound closure assays with their inability to degrade and reorganize the ECM [82]. Motile opportunist cells are able to collectively invade when the ECM is organized into tracks by fibroblasts, or enriched in collagen I, which promotes protrusion formation in both normal and mammary tumor cells [65]. Notably, activation of these hybrid EMT programs is essential for opportunist invasion [70,83,84]. 

### 3.5. Regulation of Hybrid EMT States That Promote Opportunistic Collective Invasion

The precise nature of hybrid EMT programs that confer an opportunist phenotype have begun to become unraveled. ΔNp63 is necessary for opportunistic invasion in multiple breast cancer models [83,84] and confers a hybrid EMT by directly inducing the expression of Slug and Axl [84,85]. Other EMT-TFs are not induced by ΔNp63 and E-cadherin expression is retained, possibly due to the parallel ΔNp63-mediated induction of miR205 [85]. This ΔNp63-induced hybrid EMT state is also activated in lung squamous cancer cells [84]. In models of breast ductal carcinoma in situ, ΔNp63 is activated in collectively invading cells by the recruitment of fibroblasts [85]. ΔNp63 induction is also necessary for luminal-type mammary tumor cells to invade in a genetically engineered mouse model of breast cancer [83]. These results indicate that the nature of the EMT activating signal can dictate the mode of collective invasion by being unable to induce further progression to a complete mesenchymal state. A hybrid state can also be conferred by cell lineage-specific transcription factors that restrict responses to EMT initiating signals. In this regulatory framework, loss of the restriction mechanism permits further progression towards a mesenchymal phenotype. For instance, the transcription factors GRHL2 and OVOL2 suppress Zeb1 to restrict EMT progression in lung cancer cells and promote a collective form of migration [86]. MicroRNA expression patterns also restrict EMT progression by targeting EMT-TFs and downstream mesenchymal genes that are necessary for inducing a mesenchymal state [87]. One of these mechanisms may be responsible for sustaining a hybrid state in a model of Luminal B-type breast cancer in which Snail is activated in collectively invading cells that sustain E-cadherin expression [83,88]. 

In addition to the underlying transcription regulation conferring an opportunistic hybrid state, properties of hybrid EMT cells that directly promote collective invasion have begun to be investigated. In pancreatic cancer models, a hybrid EMT state correlates with an increased storage of E-cadherin in recycling endosomes, potentially due to the increased expression of Rab11 [32]. This mechanism for promoting hybrid EMT collective invasion may be a property of breast and colon cancer cells as well [32]. Indeed, activation of ERK1/2 MAP kinases and ΔNp63 promote intracellular localization of E-cadherin in motile hybrid EMT cells, possibly through the expression of FAT2 [81,84]. KRT14 is necessary for invasion of Snail-expressing hybrid cells [83] and Axl is necessary for ΔNp63-induced invasion [85], although the specific function for these genes in this context is not known. Features of EMT programs that are necessary for single-cell invasion likely contribute to opportunist invasion if they are activated as part of a specific hybrid EMT program. However, the assigning of specific functions for these traits requires experimental confirmation. 

## 4. EMT-Induced Cells Can Influence the Invasive Properties of Siblings

### 4.1. Subpopulation Interactions That Promote Single-Cell Invasion

**Figure 3:**
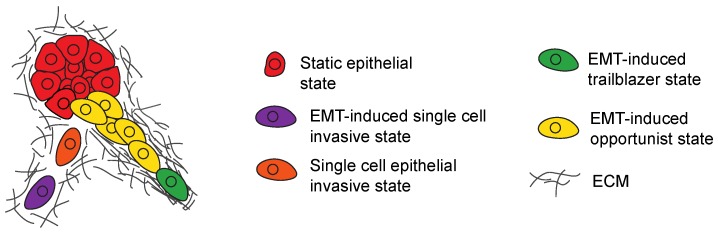
Model shows how a subpopulation of EMT induced cells can promote the invasion of siblings that lack intrinsic invasive properties. EMT induced cells (purple) can promote single cell invasion of sibling epithelial tumor cells (orange) through paracrine signaling. Cells in a trailblazer EMT state (green) can create paths in the ECM then promote the collective invasion of siblings in an opportunist EMT state (yellow).

EMT induction occurs in a fraction of cells in primary tumors [39,51,63,88,89]. In addition to conferring these cells with new invasive properties, EMT-induced cells can influence the invasive character of sibling epithelial subpopulations (Figure 3). In prostate cancer models, cells that have acquired a stable mesenchymal phenotype promote the invasion of a sibling epithelial subpopulation [90]. In this interaction, undefined secreted factors from the mesenchymal population promoted the conversion of epithelial cells into a more invasive state. This induction of single-cell invasion correlated with the activation of an EMT program in the epithelial cells, as indicated by the expression of fibronectin. The conversion of epithelial cells to a more invasive state was sustained for seven days after interacting with the mesenchymal subpopulation [90]. Consistent with this finding, cells that have undergone TGFβ-induced EMT are capable of propagating EMT induction in untreated sibling cells, which was detected by the silencing of E-cadherin [91]. Undefined paracrine signals from EMT-induced cells can also promote the invasion of neuroendocrine subpopulations in a small-cell lung cancer (SCLC) model [92]. In addition, EMT-induced cells can confer invasive properties through mechanisms that allow siblings to sustain epithelial character. Cells that have undergone an EMT in response to exogenous Twist, Snail, or Six1 expression are capable of activating a Gli1-dependent signaling pathway in epithelial cells that promotes their migration and invasion [93]. 

### 4.2. Subpopulation Interactions That Promote Collective Invasion

Trailblazer cells can promote sibling opportunist-cell invasion through a paracrine signaling independent mechanism. In this mode of interaction, paths in the ECM created by trailblazer cells promote the collective invasion of motile opportunist sibling tumor cells or normal mammary epithelial cells that lack the intrinsic capacity to initiate invasion [68]. Interestingly, paracrine signaling is not sufficient for trailblazer cells to induce the opportunist subpopulation invasion [68]. Importantly, opportunist cells are also not conferred with a trailblazer phenotype in this mode of interaction [68]. A similar type of trailblazer cell-induced invasion through path generation has been detected in lung cancer cell lines [69]. Trailblazer and opportunist cells from both breast and lung cancer populations have EMT program activation, indicating that they are in distinct EMT states [68,69]. Breast cancer trailblazer and opportunist cells express high-levels of canonical EMT-TF and vimentin, in addition to having low E-cadherin expression. Lung cancer trailblazer and opportunist populations also lack E-cadherin expression [69]. 

Genes that are specifically required for trailblazer cell invasion that were described earlier, including, DOCK10, integrin α11, DAB2, and PDGFRA, are expressed at least four-fold higher in trailblazer cells, relative to opportunist siblings [68,85]. In lung cancer populations, trailblazer cells express higher levels of VEGFA and fibronectin, both of which are required for invasion [69]. Whether these functional requirements for trailblazer-induced invasion of sibling cells are conserved across tumor types is not known. Breast cancer trailblazer and opportunist subpopulations are epigenetically distinct and the phenotypes are semi-stable, with spontaneous conversion events detected over time [68]. However, the epigenetic control mechanism itself has not been established and processes that directly control the changes in gene expression that confer the trailblazer phenotype have not been established in breast or lung cancer populations. In addition, whether signals from the microenvironment, such as TGFβ, promote a trailblazer-opportunist relationship has not been analyzed. Thus, further investigation is needed to determine the details of the mechanisms underlying these interactions and the relative contribution of the EMT-induced cells and epithelial siblings towards metastasis and treatment response.

## 5. EMT Invasion Programs Determine Metastatic Traits

### 5.1. EMT Activation Promotes Early Dissemination

Analysis of mouse models of breast and pancreatic cancer suggest that an EMT is induced in a subpopulation of cells prior to detectable primary tumor formation [94,95,96,97]. These EMT-induced cells disseminate to distant tissues [95,96] and can form up to 80% of detected metastases [97]. In a HER2/Neu amplification model, this migratory program is inactivated during tumor progression as part of a pro-growth signaling program [97]. This suggests the intriguing possibility that normal mammary tissue is more permissive to EMT induction than highly proliferative cells in primary tumors. Early tumor cell dissemination is also detected in patients with pancreatic cysts [98], although the clinical contribution of early dissemination remains largely undefined. Also, the neoplastic perturbations driving tumor growth in these genetically engineered mouse models are present throughout the epithelium, creating a greater number of potential cells that can undergo EMT. In addition, widespread oncogene may create interactions with the microenvironment that are not normally present until later in tumor development, when these genetic abnormalities are frequently acquired. Thus, the precise role of early versus late dissemination requires further evaluation. 

### 5.2. There Is EMT Program Heterogeneity in Primary Tumors

It is well-established that EMT programs are activated in invasive primary tumor cells [39,63]. In principle, EMT induction can promote the initial induction of invasion into the ECM. Consistent with this possibility, EMT program activation is sufficient to promote invasion, which triggers a transition from in situ to invasive growth in an orthotopic tumor model [85]. Moreover, trailblazer cells can induce the collective invasion of epithelial siblings in a model of ductal carcinoma in situ through a non-cell autonomous mechanism [68]. Consistent with this interaction between populations, distinct clones invade together during the initial induction of invasion in breast cancer patient tumors [99]. However, the precise point when EMT programs are activated in patient tumors remains unresolved [1]. This is, in part, due to the technical challenges of determining the timing of EMT activation with respect to occurring before or after invasion, which is impossible with current technology. Immunostaining and genetic reporters indicate that there is topographical variation with respect to EMT induction in invasive primary tumors [39,51,55,89]. The extent and nature of EMT program activation is also heterogeneous, yielding an assortment of EMT states with distinct invasive and metastatic properties [32,39,55]. This heterogeneity is influenced by clonal variability and the diversity of the tumor microenvironment [39,89]. The existence of distinct EMT states influences how and where tumor cells metastasize. In pancreatic cancer models, both hybrid and complete EMT programs are active in the same tumor. Hybrid EMT-induced cells collectively invade and disseminate as clusters of cells that specifically colonize the liver, whereas cells that have undergone a complete EMT engage in single-cell invasion and colonize the lungs [100]. In an orthotopic breast cancer model, collectively invading cells metastasize to lymph nodes while single invasive cells disseminate to the lungs [55]. Polyclonal tumor cell clusters seed lung metastases in a different set of breast cancer models [101,102], indicating that yet to be defined features of primary tumors dictate how the mode of invasion influences organotropism.

### 5.3. EMT Program Traits Influence Colonization Ability

The nature of the EMT program influences the ability of tumor cells to engage in colonizing metastatic growth. Stable and complete induction of EMTs promotes dissemination to new tissues [55,103]. However, sustained EMT induction can cause a loss of proliferative capacity and render cells dormant [55,104,105]. A mesenchymal-to-epithelial (MET) conversion after dissemination, either due to the removal of an EMT activation signal or to the induction of a reversion program, can re-initiate growth and promote colonization [103,106]. There are potential alternatives to the EMT-MET reversion mechanism for metastasis. Hybrid EMT states confer invasive properties while allowing cells to retain intrinsic metastatic growth potential [39,107,108]. In addition, cells that have acquired a mesenchymal phenotype can promote metastasis of a second population that lacked stable EMT features at the time of injection into the mouse in breast, prostate, and SCLC tumor models [90,92,93]. Notably, the cells that had undergone a stable EMT did not form metastases, highlighting the potential importance of non-cell autonomous mechanisms in promoting dissemination and colonization [90,92,93]. 

## 6. Conclusions

Extensive investigation using an array of tumor models supported by patient tumor analysis has demonstrated that EMT programs contribute to tumor invasion and metastasis. The basic features of EMT programs that control invasion have been established. More recently, the diversity of EMT programs and the phenotypes they induce during tumorigenesis have been recognized. Building upon these discoveries, there are a number of challenges that must be addressed to understand the regulation and function of EMTs with the goal of improving cancer patient diagnosis and treatment. It is critical to determine at which point EMTs are induced during tumor progression to define precisely how EMTs influence metastasis. The contributions of EMTs towards metastasis has largely relied on models in which tumor cells have progressed to a near fully mesenchymal state. However, hybrid states are frequently detected in primary tumors and may be the predominant type of EMT [109]. Yet the processes that confer hybrid EMTs and the functional requirements for hybrid cells to metastasize are unknown. How variability in EMT states present in a tumor contribute to metastasis has begun to be appreciated and requires further investigation. In particular, whether a specific subset of EMT states influences metastasis and if distinct EMT states create synergistic relationships that contribute to metastasis should be determined. Notably, certain transcription factors or EMT state-specific components may not be involved in metastatic events, but may be necessary for other features, such as acquired resistance to chemotherapy [110,111,112,113]. Thus, it is essential to define the composition of EMT signaling networks that are active in vivo and to determine their precise functions in promoting metastasis. Finally, any new analysis of EMTs should consider cell autonomous and non-cell autonomous functions, which have begun to be recognized, however lack a detailed mechanistic understanding.
